# Supplementary material for: Nectin Cell Adhesion Molecule 4 (NECTIN4) Expression in Cutaneous Squamous Cell Carcinoma: A New Therapeutic Target?
Source: Biomedicines. 2021 Mar 30;9(4):355. doi: 10.3390/biomedicines9040355 (PMC8067104; doi:10.3390/biomedicines9040355)
Supplement: Supplementary file 1 [file biomedicines-09-00355-s001.zip › biomedicines-1155716-supplementary.pdf]

Supplementary Materials

Nectin cell adhesion molecule 4 (NECTIN4) expression in cutaneous squamous cell carcinoma: A new therapeutic target?

Yuka Tanaka, Maho Murata, Yoshinao Oda, Masutaka Furue, Takamichi Ito

Corresponding author: Takamichi Ito, Department of Dermatology, Graduate School of Medical Sciences, Kyushu University, 3-1-1 Maidashi, Higashi-ku, Fukuoka City, Fukuoka, 812-8582, Japan

Tel.: +81-92-642-5585, Fax: +81-92-642-5600

E-mail address: takamiti@dermatol.med.kyushu-u.ac.jp

This file contains:

Supplementary Figure S1

Supplementary Tables S1–2

Supplementary Figure S1

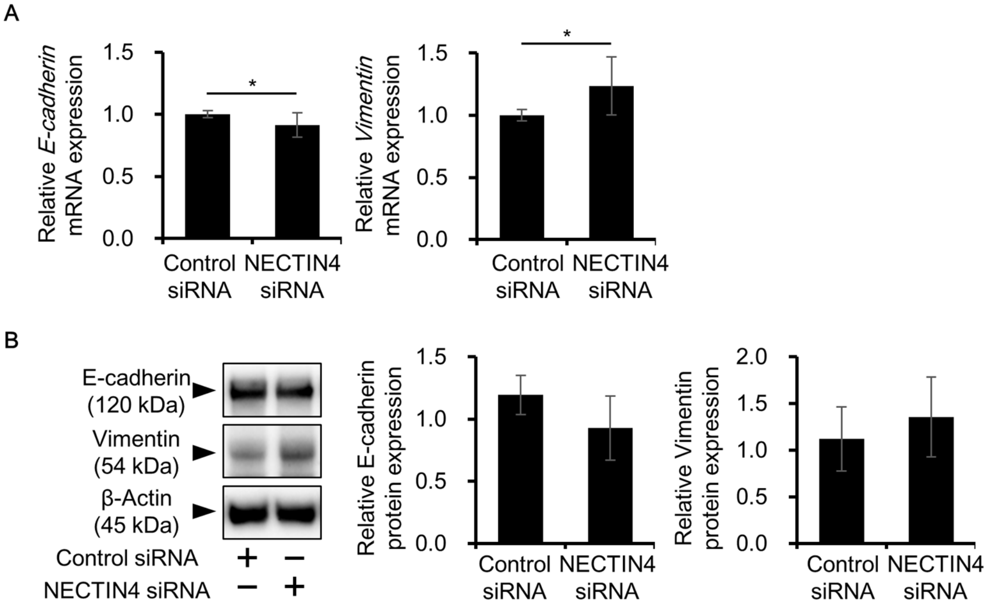

Supplementary Figure S1. Effect of NECTIN4 inhibition on E-cadherin and vimentin expression.

(**A**) A431 cells were transfected with control or NECTIN4 siRNA for 48 h and assessed for E-cadherin or vimentin mRNA (**A**) and protein (**B**) expression after 48 and 72 h, respectively. Representative blot images are shown (**B**). Data are the mean ± SD of three independent experiments. * $p < 0.05$.

Supplementary Table S1: Primer sequences for qRT-PCR

Gene Symbol		Sequence
Cyclin D1	Sense	5′-GCTGCGAAGTGGAAACCATC-3′
	Antisense	5′-CCTCCTTCTGCACACATTTGAA-3′
ZEB1	Sense	5′-GCACCTGAAGAGGACCAGAG-3′
	Antisense	5′-TGCATCTGGTGTTCCATTTT-3′
ZEB2	Sense	5′-TTTCAGGGAGAATTGCTTGA-3′
	Antisense	5′-CACATGCATACATGCCACTC-3′
SNAIL	Sense	5′-GCCTAGCGAGTGGTTCTTCT-3′
	Antisense	5′-TAGGGCTGCTGGAAGGTAAA-3′
NECTIN4	Sense	5′-CAAAATCTGTGGCACATTGG-3′
	Antisense	5′-GCTGACATGGCAGACGTAGA-3′
β-actin	Sense	5′-ATTGCCGACAGGATGCAGA-3′
	Antisense	5′-GAGTACTTGCGCTCAGGAGGA-3′

Supplementary Table S2: Specifics of antibodies for western blotting

Name of antibody	Host	Manufacturer	Catalog no (RRID)	Dilutions
Anti-human NECTIN4	Rabbit	Abcam	ab192033	1:1,000
Anti-human ZEB1	Rabbit	Abcam	ab155249	1:1,000
Anti-human ZEB2	Rabbit	Abcam	ab138222 (AB_2801551)	1:500
Anti-human ACTB	Rabbit	Cell Signaling Technologies	#4970 (AB_223172)	1:2,000
Anti-Akt	Rabbit	Cell Signaling Technologies	#9272 (AB_329827)	1:1,000
Anti-pAkt	Rabbit	Cell Signaling Technologies	#4969 (AB_2315049)	1:2,000
Anti-ERK	Rabbit	Cell Signaling Technologies	#9102 (AB_330744)	1:1,000
Anti-pERK	Rabbit	Cell Signaling Technologies	#4370 (AB_2315112)	1:2,000
Anti-human cyclin D1	Rabbit	Cell Signaling Technologies	#55506 (AB_28227374)	1:1,000
Anti-snail	Rabbit	Cell Signaling Technologies	#3879 (AB_2255011)	1:500
Anti-rabbit IgG horseradish peroxidase	Goat	Cell Signaling Technologies	#7074 (AB_2099233)	1:10,000
